# Introduction to the Toxins Special Issue on Identification and Functional Characterization of Novel Venom Components

**DOI:** 10.3390/toxins12050336

**Published:** 2020-05-20

**Authors:** Steven D. Aird

**Affiliations:** Technical Editor, Nakama, Onna-son, Kunigami-gun, Okinawa-ken 904-0401, Japan; sda.tech.ed@icloud.com

Throughout most of the 20th century, the toxinological literature consisted largely of pharmacological and functional characterizations of crude venoms and venom constituents, often constituents that could not be identified unambiguously. The advent of amino acid composition analysis in the 1950s enabled the first forays into physical characterizations of purified toxins, although these remained few in number until the 1970s. Then, tryptic and chymotryptic cleavage of venom proteins coupled with manual Edman degradation began to provide the first complete sequences, particularly of three-finger toxins. Polyacrylamide gel electrophoresis and superior resins for liquid chromatography permitted improved purification and better gross structural characterization of venom components. The early 1980s saw the advent of automated Edman degradation, and entire sequences of longer proteins began to appear in the literature. Then, the molecular biology revolution made it possible to generate cDNA sequences of more and larger proteins, followed by mass spectrometry-based proteomics and quantitative high-throughput DNA sequencing and genomics. Today, we face a hitherto unprecedented situation in which our capacity to generate sequence/structural data has completely overwhelmed our capacity to characterize venom constituents functionally. This Special Issue of *Toxins* comprises 11 publications addressing the discovery and functional characterization of novel venom constituents of vertebrate and invertebrate venoms.

Tadokoro et al. [1] review the functional diversity of snake toxins belonging to the Cysteine-Rich Secretory Protein (CRISP) superfamily and find that despite their broad taxonomic distribution, very few of these proteins have been functionally characterized; however, those that have, exhibit diverse activities, inhibiting ion channels and angiogenesis, increasing vascular permeability, and promoting inflammatory responses (leukocyte and neutrophil infiltration).

Pérez-Peinado et al. [2] review snake toxins that have been exploited or are being explored for diagnosis and treatment of various cardiovascular disorders and blood abnormalities. They focus on development of antimicrobial and anticancer drugs, reviewing their principal activities in vitro and in vivo, their structures, mechanisms of action, and their potential as drug leads.

Santibanez-Lopez et al. [3] use new and previously published transcriptomic resources to assess evolutionary relationships of closely related scorpions from the family Hadruridae and their toxins. In particular, they survey potassium channel toxins known as scorpine-like peptides, showing that these peptides exhibit gene duplications. The authors find that more toxin sites are evolving under negative selection than under positive selection.

Delgado-Prudencio et al. [4] examine the means by which many scorpion venom peptides are amidated at their C-termini, a post-translational modification that is essential for correct biological functioning of these peptides. They report the existence of a dual enzymatic α-amidation system, and identify the enzymes in scorpion venom glands responsible.

Ullah et al. [5] examine the structure of snake venom phosphodiesterases, little studied enzymes responsible for unleashing purine nucleosides in the prey to promote hypotension, inhibition of platelet aggregation, edema, and paralysis. They examine the enzyme from *Crotalus adamanteus* venom and report that this PDE comprises a somatomedin B domain, a somatomedin B-like domain, an ectonucleotide pyrophosphatase domain, and a DNA/RNA non-specific domain. They find that snake venom PDEs exhibit high sequence identity, but comparatively low identity with mammalian and bacterial PDEs. Nonetheless, they suggest that intraspecific sequence differences may account for different substrate specificities.

Inamaru et al. [6] follow up on earlier work to report a novel, sixth small serum protein from *Protobothrops flavoviridis*. Using comprehensive genomic analysis, the authors conclude that some SSPs were present in all snake genomes before the divergence of venomous snakes, but that others arose specifically in venomous snakes. The authors report that the evolutionary emergence of SSP genes is probably related to physiological functions of SSPs and that venom SSP composition may reflect snake habitat, prey, and venom differences.

Kuo et al. [7] examine mechanisms and structure–activity relationships of disintegrins TFV-1 and TFV-3, from *Protobothrops flavoviridis* venom, which have very different pro-hemorrhagic tendencies. The find that TFV-1 selectively inhibits Gα13-mediated platelet aggregation without affecting physiological hemostatic processes. It causes neither severe thrombocytopenia nor bleeding in mice and does not induce hypocoagulation in human whole blood. In contrast, TFV-3 and eptifibatide exhibit all of these hemostatic effects. 

Heep et al. [8] explore the peptidome of a predatory ant, *Manica rubida*, and observe severe fitness costs in the pea aphid (*Acyrthosiphon pisum*), a common agricultural pest. They identify a novel decapeptide, which, while inactive against bacteria and fungi, reduces aphid survival and reproduction. Both crude venom and the peptide reversibly paralyze injected aphids, inducing loss of body fluids. They suggest that this venom may be a promising source of additional bio-insecticide leads.

Huancahuire-Vega et al. [9] report the purification and biochemical and functional characterization of ACP-TX-I and ACP-TX-II, two phospholipases A_2_ (PLA_2_) from *Agkistrodon contortrix pictigaster* venom. ACP-TX-I is a monomeric, catalytically inactive K49 PLA_2_, exhibiting markedly diminished myotoxic and inflammatory activity, compared to dimeric K49 toxins. ACP-TX-II is an enzymatically active D49 PLA2, exhibiting in vivo local myotoxicity, edema-forming activity, and in vitro cytotoxicity. ACP-TX-I PLA2 is likewise cytotoxic, indicating that cytotoxicity does not require enzymatic activity.

Tourki et al. [10] describe Lebetin 2 (L2), a natriuretic peptide from *Macrovipera lebetinus* venom and report that following myocardial infarction, L2 reduces leukocyte and proinflammatory M1 macrophage infiltration into the infarcted area. It also increased anti-inflammatory M2-like macrophages, inducing a higher density of endothelial cells and cardiomyocytes than BNP. The authors find that L2 has strong, acute, prolonged cardioprotective effects that reduce ischemic reperfusion-induced necrotic and fibrotic effects.

Wu et al. [11] describe the isolation of a novel bradykinin-related peptide from the skin secretion of a ranid frog, *Ordorrana hejiangensis*. They report that when Arginine-4 is substituted with Leucine, this bradykinin agonist is transformed into a bradykinin antagonist.

## References

[1] Tadokoro T., Modahl C.M., Maenaka K., Aoki-Shioi N. (2020). Cysteine-Rich Secretory Proteins (CRISPs) from Venomous Snakes: An Overview of the Functional Diversity in a Large and Underappreciated Superfamily. Toxins.

[2] Pérez-Peinado C., Defaus S., Andreu D. (2020). Hitchhiking with Nature: Snake Venom Peptides to Fight Cancer and Superbugs. Toxins.

[3] Santibáñez-López C.E., Graham M.R., Sharma P.P., Ortiz E., Possani L.D. (2019). Hadrurid Scorpion Toxins: Evolutionary Conservation and Selective Pressures. Toxins.

[4] Delgado-Prudencio G., Possani L.D., Becerril B., Ortiz E. (2019). The Dual α-Amidation System in Scorpion Venom Glands. Toxins.

[5] Ullah A., Ullah K., Ali H., Betzel C., ur Rehman S. (2019). The Sequence and a Three-Dimensional Structural Analysis Reveal Substrate Specificity among Snake Venom Phosphodiesterases. Toxins.

[6] Inamaru K., Takeuchi A., Maeda M., Shibata H., Fukumaki Y., Oda-Ueda N., Hattori S., Ohno M., Chijiwa T. (2020). Discovery of the Gene Encoding a Novel Small Serum Protein (SSP) of *Protobothrops flavoviridis* and the Evolution of SSPs. Toxins.

[7] Kuo Y.-J., Chung C.-H., Pan T.-Y., Chuang W.-J., Huang T.-F. (2020). A Novel α_IIb_β_3_ Antagonist from Snake Venom Prevents Thrombosis without Causing Bleeding. Toxins.

[8] Heep J., Skaljac M., Grotmann J., Kessel T., Seip M., Schmidtberg H., Vilcinskas A. (2019). Identification and Functional Characterization of a Novel Insecticidal Decapeptide from the Myrmicine Ant *Manica rubida*. Toxins.

[9] Huancahuire-Vega S., Hollanda L.M., Gomes-Heleno M., Newball-Noriega E.E., Marangoni S. (2019). ACP-TX-I and ACP-TX-II, Two Novel Phospholipases A_2_ Isolated from Trans-Pecos Copperhead *Agkistrodon contortrix pictigaster* Venom: Biochemical and Functional Characterization. Toxins.

[10] Tourki B., Dumesnil A., Belaidi E., Ghrir S., Godin-Ribuot D., Marrakchi N., Richard V., Mulder P., Messadi E. (2019). Lebetin 2, a Snake Venom-Derived B-Type Natriuretic Peptide, Provides Immediate and Prolonged Protection against Myocardial Ischemia-Reperfusion Injury via Modulation of Post-Ischemic Inflammatory Response. Toxins.

[11] Wu Y., Shi D., Chen X., Wang L., Ying Y., Ma C., Xi X., Zhou M., Chen T., Shaw C. (2019). A Novel Bradykinin-Related Peptide, RVA-Thr6-BK, from the Skin Secretion of the Hejiang Frog; Ordorrana hejiangensis: Effects of Mammalian Isolated Smooth Muscle. Toxins.

